# CXCL8 Knockout: A Key to Resisting *Pasteurella multocida* Toxin-Induced Cytotoxicity

**DOI:** 10.3390/ijms25105330

**Published:** 2024-05-14

**Authors:** Jianlin Yuan, Qin Zhao, Jinfeng Li, Yiping Wen, Rui Wu, Shan Zhao, Yi-Fei Lang, Qi-Gui Yan, Xiaobo Huang, Senyan Du, San-Jie Cao

**Affiliations:** 1 Research Center for Swine Disease, College of Veterinary Medicine, Sichuan Agricultural University, Chengdu 611130, China; yuanjianlin715@163.com (J.Y.); zhao.qin@sicau.edu.cn (Q.Z.); sicau961013@163.com (J.L.); wyp@sicau.edu.cn (Y.W.); wurui1977@sicau.edu.cn (R.W.); zhaoshan419@163.com (S.Z.); y_langviro@163.com (Y.-F.L.); yqg@sicau.edu.cn (Q.-G.Y.); huangxiaobo@sicau.edu.cn (X.H.); 2 Sichuan Science-Observation Experimental Station of Veterinary Drugs and Veterinary Diagnostic Technique, Ministry of Agriculture and Rural Affairs, Chengdu 611130, China; 3 National Demonstration Center for Experimental Animal Education, Sichuan Agricultural University, Chengdu 611130, China

**Keywords:** *Pasteurella multocida* toxin, CXCL8, PK15 cells, apoptosis

## Abstract

*Pasteurella multocida*, a zoonotic pathogen that produces a 146-kDa modular toxin (PMT), causes progressive atrophic rhinitis with severe turbinate bone degradation in pigs. However, its mechanism of cytotoxicity remains unclear. In this study, we expressed PMT, purified it in a prokaryotic expression system, and found that it killed PK15 cells. The host factor CXCL8 was significantly upregulated among the differentially expressed genes in a transcriptome sequencing analysis and qPCR verification. We constructed a CXCL8-knockout cell line with a CRISPR/Cas9 system and found that CXCL8 knockout significantly increased resistance to PMT-induced cell apoptosis. CXCL8 knockout impaired the cleavage efficiency of apoptosis-related proteins, including Caspase3, Caspase8, and PARP1, as demonstrated with Western blot. In conclusion, these findings establish that CXCL8 facilitates PMT-induced PK15 cell death, which involves apoptotic pathways; this observation documents that CXCL8 plays a key role in PMT-induced PK15 cell death.

## 1. Introduction

*Pasteurella multocida* (*P. multocida*) is a facultative anaerobic Gram-negative bacillus that has five serogroups, A, B, D, E, and F, according to differences in its capsular antigens [1,2]. Its most important virulence factors are the capsule, outer membrane protein, lipopolysaccharide, *P. multocida* toxin (PMT), fimbriae, and adhesin molecules [3,4]. *P. multocida* infection is a zoonotic disease that can infect many kinds of wild and domestic animals, causing diseases such as fowl cholera, bovine hemorrhagic septicemia, zoonotic pneumonia, and swine atrophic rhinitis [5,6,7]. These diseases can spread to humans through animal scratches, leading to wound abscesses and meningitis [8].

PMT, a 146 kDa heat-labile toxin, is only secreted by some serogroups A and D toxigenic strains encoded by the *toxA* gene [9]. This protein belongs to the skin necrosis toxin family and is one of the intracellularly acting, multipartite A-B toxins. The PMT toxin mainly comprises an N-terminal domain and C-terminal domain [10]. The N-terminal domain is mainly responsible for binding with the host cell receptor that promotes the transport of the toxic-activity C-terminal domain by cells to the cytoplasm, and then the release of the C-terminal toxic region [11,12], thereby stimulating relevant signal pathways in cells to cause cytotoxicity [13]; recent studies have shown that LRP1 is one of the cell surface receptor of PMT, which promotes the cytotoxicity of PMT [14,15]. Phospholipid binding mainly activates heterotrimeric G proteins of the α subunits to activate intracellular multiple signaling pathways [16,17], including mTORC1 and ERK activation and PLC-β-mediated phosphoinositide turnover; most of the cascade reactions of these signal pathways promote cell proliferation [18,19,20]. Previous research focused on PMT-induced cell proliferation [21]. As the most potent mitotic protein, PMT can promote Swiss 3T3 cell proliferation [22], stimulate osteoclast differentiation, and promote cell proliferation [23,24]. PMT can also stimulate the anti-apoptotic pathways of HEK293 cells by activating the G protein receptor pathway [25] and the activity of rat superior cervical ganglion (SCG) neurons and NG108-15 neuronal cells [26]. PMT activation of the PI3K-AKT and RhoA pathways stimulates cell proliferation and differentiation [27,28]. The protein encoded by the *CXCL8* gene is a member of the CXC chemokine family; it can activate the PI3K, PKC, and Rho signal pathways [29,30,31], just as the PI3K-AKT signaling pathways was activated, further activating IKK and NF-κB that promote efficient expression of CXCL8 [32]. These are like the cell signal pathway activated by PMT. In addition, CXCL8 is critical for activating and transporting inflammatory mediators and promoting tumor growth and metastasis. CXCL8, by binding to CXCR1/2, triggers a cascade of downstream signals to regulate cell proliferation and death [33,34]. The encoded protein CXCL8 is commonly referred to as interleukin-8 (IL-8). The binding of IL-8 to one of its receptors (IL-8RB/CXCR2) increases the permeability of blood vessels, and increasing levels of IL-8 are positively correlated with the increased severity of multiple disease outcomes [35,36]. CXCL8 is mainly expressed in tumor cells as a marker gene for tumor healing [37]. CXCL8 is also expressed in macrophages, epithelial cells, and other cells [38]. When cells are not stimulated, CXCL8 is mainly not expressed. Once stimulated, the cells will express CXCL8 [39]. CXCL8 expression is affected by IFNγ, TNFα, and NF-κB [40,41].

It is well known that PMT is the causative factor of porcine atrophic rhinitis [42]. Previous studies have also shown that non-oral administration of PMT to pigs can lead to liver sclerosis, renal failure, and a significant and persistent decrease in peripheral blood lymphocytes, as well as growth retardation [43,44]. In addition, PMT causes hepatotoxicity in rats and myocardial fibrosis in mice [45,46]. When the toxin-producing strain grows in the nasal cavity of animals, PMT may directly invade the nasal turbinate. However, based on its ability to invade the entire body, the toxin may also affect the tonsils or other anatomical sites where bacteria settle [47]. In pigs, immunohistochemical investigations of 30 cases of porcine dermatitis and nephropathy syndrome after a short clinical illness revealed *P. multocida*-specific staining in 26 of the cases, primarily in the renal tubular epithelial cells of the proximal convoluted tubules, but also in the glomeruli, in lesions of renal vasculitis, and the cytoplasm of interstitial mononuclear cells [48]. In human beings, a case has been reported of cat scratch-induced *P. multocida* infection, presenting with disseminated intravascular coagulation and acute renal failure [49]. However, there is still relatively little research on the nephrotoxicity of PMT, especially in selecting and using renal cell toxicity models. PK15 is a cell line exhibiting an epithelial morphology that was isolated from the kidney of an adult pig, and PK15 cells have become a commonly used model for nephrotoxicity research [50,51,52]. However, the role of CXCL8 has not been clarified in PK15 cells, and the relationship between CXCL8 and PMT has not been reported. This report presents the first transcriptome sequencing analysis of PK15 cells, demonstrating that *CXCL8* gene deletion protects against PMT-induced apoptosis in PK15 cells, which lays the foundation for further research on the mechanism of CXCL8-mediated PMT-induced apoptosis.

## 2. Results

### 2.1. PMT and PMT-C1165S Recombinant Protein

**Figure 1:**
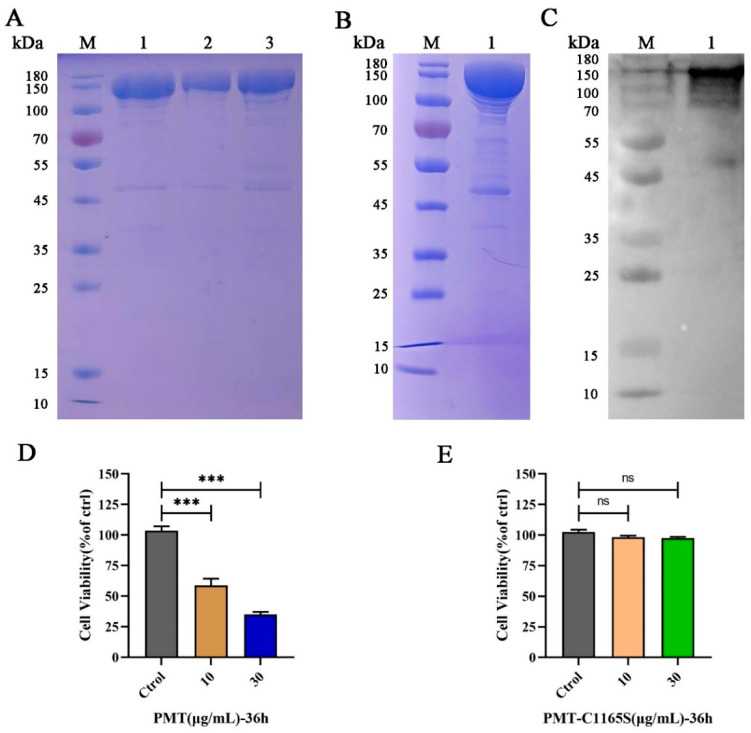
The expression and identification of PMT and PMT-C1165S. (**A**) The expression and purification of PMT. M, protein marker; line 1–3, recombinant His-tagged PMT expressed in Escherichia coli and purified through Ni-NTA and gel filtration columns. (**B**) Dialysis and concentration of PMT. M, protein marker; line 1, recombinant His-tagged PMT. (**C**) Western blot identification of PMT protein. M, protein marker; line 1, recombinant His-tagged PMT. (**D**) Cell viability assay of PK15 cells by CCK-8 after incubation with 10 and 30 μg/mL of PMT for 36 h. (**E**) Cell viability assay of PK15 cells by CCK-8 after incubation with 10 and 30 μg/mL of PMT-C1165S for 36 h. Data are represented as means ± S.D. *n* = 3. ns > 0.05, *** *p* < 0.001.

Recombinant *ToxA* genes expressing the PMT protein were confirmed by sequence analysis (Figure S1A). The recombinant plasmid pCold I-toxA was constructed by sequencing (Figure S1B) and transformed into BL21 (DE3), which expressed the 146 kDa His-tagged PMT toxin protein (Figure 1A, line 1–3). Further dialysis and concentration removed imidazole and endotoxin, and the results of the SDS-PAGE for the PMT protein were compared (Figure 1B, line 1). An exposure stripe on the Western blotting corresponded with the monoclonal anti-His line (Figure 1C, line 1); in the results of the SDS-PAGE for the PMT mutant without catalytic activity (PMT-C1165S) (Figure S1C, line 1–3), a Western blotting analysis showed the expression of PMT-C1165S (Figure S1D, line 1–3); this demonstrated that PMT and PMT-C1165S were correctly expressed.

### 2.2. PMT Induces PK15 Cell Death

To verify the cytotoxicity of the PMT and PMT-C1165S toxin protein for PK15 cells, we treated the cells with various concentrations of PMT and PMT-C1165S protein at various times. After 24 h of PMT stimulation, cells in the 30 μg/mL treatment group showed a slight swelling compared with those of the control group; after 36 h stimulation, obvious cytotoxic effects were observed. After treatment with PMT at concentrations of 10 μg/mL and 30 μg/mL, a series of morphological changes were observed. Initially, the cells became round and exhibited increased brightness. Subsequently, a subset of cells detached from the culture dish and started floating freely in the medium. Furthermore, the nucleus within the cells underwent significant swelling. Ultimately, the integrity of the cell membranes was compromised, leading to their rupture and eventual cell death (Figure S2A). Moreover, CCK-8 results revealed that the toxic effect of PMT on PK15 cells was increased with increasing PMT concentration after 36 h treatment (Figure 1D). However, there is no cytotoxicity after treatment with PMT-C1165S at concentrations of 10 μg/mL and 30 μg/mL (Figure 1E and Figure S2B). The IC50 of PK15 cells treated with PMT for 36 h was 9.32 μg/mL (Figure S2C). This result indicates that the PMT toxin protein induces PK15 cell death, although the mechanism of cell death was not explained.

### 2.3. Quality Control and Mapping of RNA Sequencing Results

Four transcriptome libraries were constructed from PK15- and PMT-treated cell samples, to identify the essential host factor for PMT-induced PK15 cell death in the transcriptome profile. RNA sequencing revealed nearly 195 million raw reads; after quality control, about 194 million clean reads remained. Thus, the proportion of clean data was more than 99% for each sample. For all samples, the Q20 and Q30 percentages of clean data were more than 98% and 94%, respectively, and the GC content of clean reads was more than 46.66% (Table 1). These findings indicated that the clean reads were high quality and suitable for further analysis.

**Figure 2:**
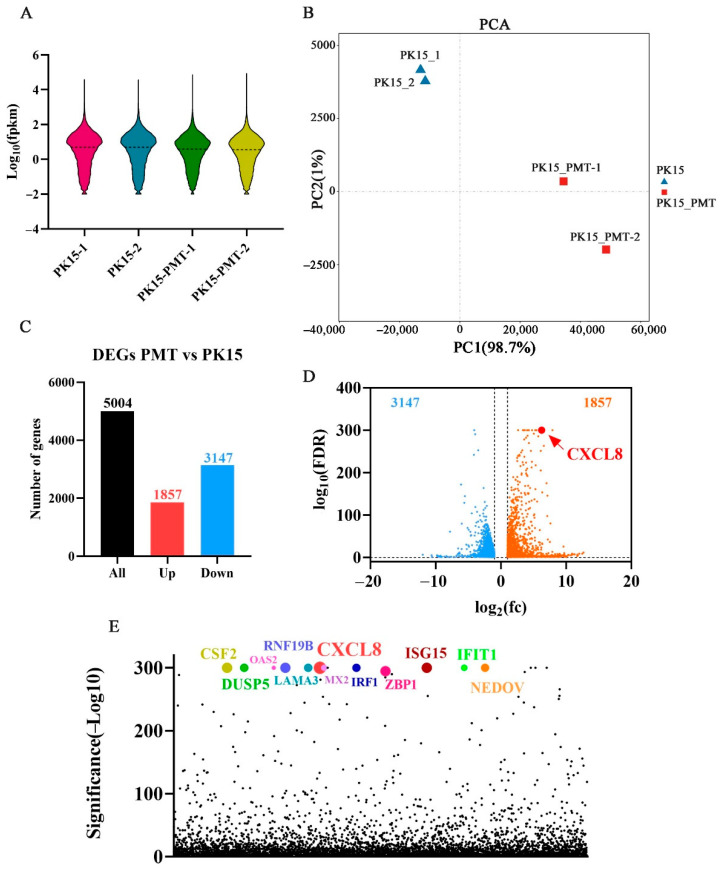
RNA-Seq analysis of differentially expressed genes (DEGs) in PMT treated cells. (**A**) Violin plot of gene expression patterns for each sample, with dotted line representing median. (**B**) Principal component analysis (PCA) of four WT- or PMT-treated PK15 cells samples. (**C**) Numbers of upregulated and downregulated DEGs of PK15 cell with PMT treatment. (**D**) Volcano plot showing DEGs of PK15 cell with PMT treatment. (**E**) Scatter plot of *p* value showing DEGs of PK15 cell with PMT treatment. Dots in various colors represent the top 12 genes.

The clean reads were aligned with the pig ribosome database, and the ribosome reads were removed when mismatches were not allowed; the retained unmapped reads were used for further analysis. As shown in Table 2, the unique mapped reads of the four samples ranged from 88.13% to 90.11%. In addition, more than 61.64% of the mapped sequences were located in the exonic region, while the percentages of intronic and intergenic sequences ranged from 22.21% and 5.08% to 31.70% and 6.80%, respectively. An average of 15,018 expressed genes and 910 novel genes were identified in the four samples (Table 2). The gene expression distribution of the samples, based on the FPKM value of each gene, is displayed through the expression distribution map (Figure 2A). A Pearson’s correlation analysis and principal component analysis were carried out based on the expression level in each sample (Figure 2B). The PK15 and PMT treatment groups were divided into two distinct regions, with similar samples clustered together.

### 2.4. Transcriptome Sequencing Analysis of Differentially Expressed Genes

A total of 5004 differentially expressed genes (DEGs) were screened out by comparing control and PMT-treatment cells. According to the standard of a false discovery rate (FDR) ≤ 0.05, among the DEGs, 1857 and 3147 genes were up- and downregulated, respectively (Figure 2C). Combined with the values analysis of log2(fc) and false discovery rate (FDR), CXCL8 (C-X-C motif chemokine ligand 8) ranks first among the DEGs (Figure 2D). According to the significance analysis of the *p* value, CXCL8 still ranks top among the DEGs (Figure 2E).

**Figure 3:**
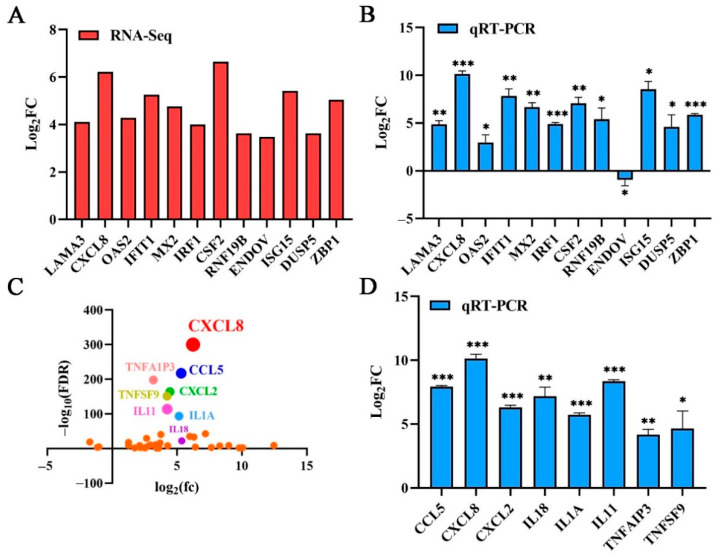
Confirmation of transcriptome sequencing data by qRT-PCR. (**A**) The top 12 genes ranked according to the significance of differences and their log values of FC. (**B**) The top 12 genes were selected for qRT-PCR verification; these DEGs’ expression levels were estimated using the 2^−ΔΔCT^ method. (**C**) DEGs related to immune and inflammatory responses were selected. Dots in various colors represent the top eight genes. (**D**) Confirmation of transcriptome sequencing data by qRT-PCR. DEGs related to immune and inflammatory responses were selected randomly for qRT-PCR analysis, and the expression levels of those DEGs were estimated with the 2^−ΔΔCT^ method. Data are represented as means ± S.D.; *n* = 3. * *p* < 0.05; ** *p* < 0.01; *** *p* < 0.001.

According to the *p* value significance analysis results, the top 12 genes (*CXCL8*, *LAMA3*, *OAS2*, *IFIT1*, *MX2*, *IRF1*, *CSF2*, *RNF19B*, *ENDOV*, *ISG15*, *DUSP5*, and *ZBP1*) were selected (Figure 2E and Figure 3A). The transcriptional level was preliminarily verified by qRT-PCR.

### 2.5. Validation of Transcriptomic Data Using qRT-PCR

Based on the results of the RNA sequencing, twelve genes were selected for qRT-PCR and further analysis to verify their expression profiles. The qRT-PCR results of the 11 genes were consistent with the RNA sequencing results, except for the *ENDOV* gene. PK15 without the PMT toxin was used as the control for normalization, the *GAPDH* gene serving as an internal control. The upregulation of the *CXCL8* gene was the most significant among them (Figure 3A,B). The result verified the reliability of the RNA sequencing data. As a cellular chemokine, CXCL8 participates in various cell signaling pathways. Therefore, combined with the values analysis of log2(fc) and false discovery rate (FDR), the cytokines interleukin, interferon, chemokine, and the tumor necrosis factor in the transcriptome sequencing results for significant differences, the *CXCL8* gene was ranked first (Figure 3C). We selected the top eight cytokines, (CXCL8, CCL5, CXCL2, IL1A, IL18 TNFAIP3, TNFSF9, and IL11) for transcription verification. Under PMT stimulation, the cytokines were significantly upregulated (Figure 3D), confirming that the RNA sequencing data were accurate. The transcription level of the *CXCL8* gene was most significantly upregulated, with an upregulation of more than 800-fold (Figure 3D). Therefore, we selected the *CXCL8* gene for further study.

### 2.6. CXCL8 Facilitates PMT-Induced Cell Death

**Figure 4:**
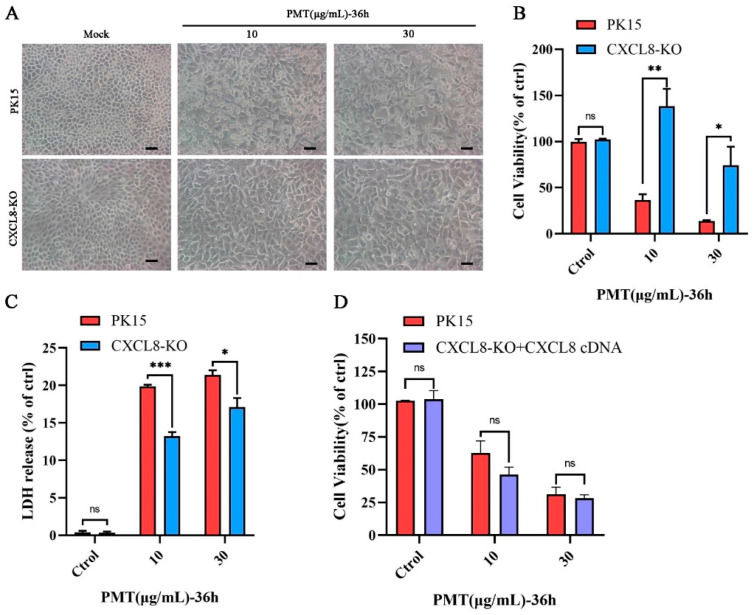
Assessing the sensitivity of CXCL8 knockout cells to PMT. (**A**) Representative micrographs of PK-15 and CXCL8-KO cell lines after incubation with PMT (36 h, 37 °C). Scale bar, 100 μm. (**B**) Cell viability assay of PK15 and CXCL8-KO cells by CCK-8 after incubation with PMT. (**C**) LDH release of PK15 and CXCL8-KO cells was detected after incubation with PMT. (**D**) Cell viability assay of CXCL8-KO cells and CXCL8 complementation cells by CCK-8 after incubation with PMT. Data are represented as means ± S.D.; *n* = 3. ns > 0.05, * *p* < 0.05; ** *p* < 0.01; *** *p* < 0.001.

The CXCL8-knockout PK15 cell line was constructed using the CRISPR/Cas9 system. DNA sequencing confirmed that the *CXCL8* gene caused a frameshift mutation by the two sgRNAs targeted (Figure S3A,B). The transcription level of the *CXCL8* gene in knockout cells was detected with qRT-PCR; the transcription level was significantly reduced (Figure S3C). In addition, compared with normal PK15 cells, the expression level of CXCL8 was significantly reduced in knockout cells according to the swine CXCL8 ELISA kit (Figure S3D). It indicated that the gene was successfully knocked out. Compared with normal PK15 cells, CXCL8 knockout cells considerably decreased susceptibility to cytotoxicity PMT-induced PK15 cell death (Figure 4A). Moreover, the results of the CCK8 assay revealed that CXCL8 knockout cells were resistant to PK15 cytotoxicity induced by PMT (Figure S3E). However, CXCL8 knockout cells were less resistant when the concentrations of PMT were increased, compared to the PK15 cells; CXCL8 knockout cells began to die when PMT was at a concentration of 30 μg/mL (Figure 4B), and the release of LDH was also significantly inhibited; with concentrations of PMT increasing, the release of LDH gradually increases in CXCL8 knockout cells. Compared to the PK15 cells, LDH release is more affected at lower concentrations of PMT (Figure 4C). In addition, it was found that the CXCL8 cDNA replenishment of CXCL8 KO cells recovered the toxicity of PMT (Figure 4D). Overall, the *CXCL8* gene mediates PMT-induced PK15 cell death. However, the specific death mechanism needs to be studied.

### 2.7. CXCL8 Knockout Decreases PMT-Induced Cell Apoptosis

**Figure 5:**
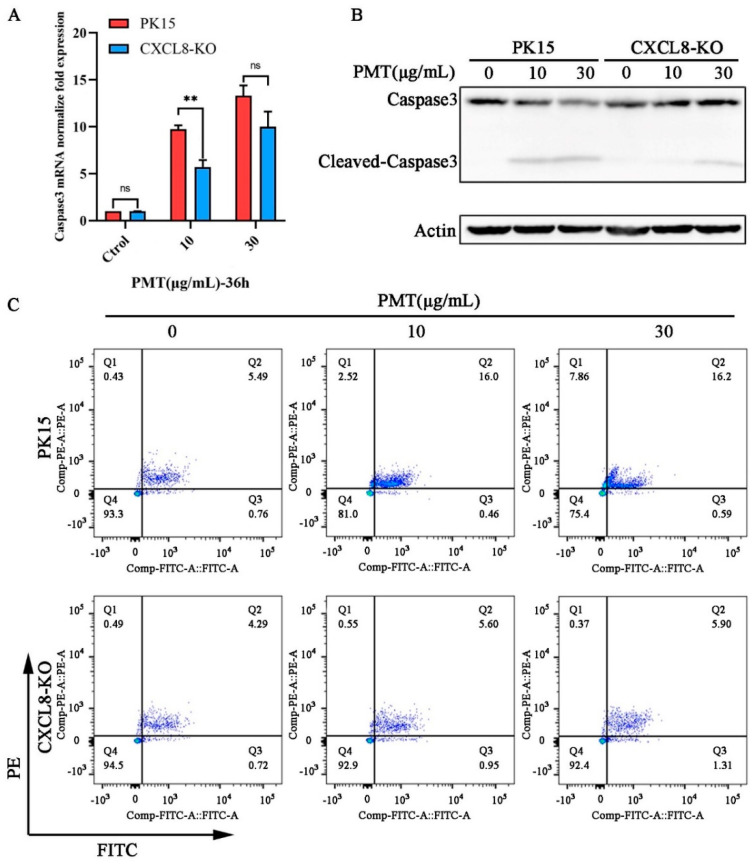
CXCL8 mediates PMT-induced apoptosis in PK15 cells. (**A**) The mRNA level of Caspase3 in PK15 and CXCL8-KO cells was detected with qRT-PCR after incubation with PMT. Data are represented as means ± S.D.; *n* = 3. ns > 0.05, ** *p* < 0.01. (**B**) Immunoblot analysis of Caspase-3-cleaved protein extracts from PK15 normal cells and CXCL8-KO cells after incubation with PMT (36 h, 37 °C). (**C**) Flow cytometry detection of apoptosis rate in PK15 and CXCL8-KO cells under PMT stimulation. Q1, the percentage of necrotic cells; Q2, late the percentage of apoptotic cell; Q3, early the percentage of apoptotic cell; Q4, the percentage of normal cells.

In a further exploration of the pathway of *CXCL8* gene-mediated PMT-induced PK15 cell death, qRT-PCR revealed that the transcription level of the apoptosis marker gene Caspase3 was decreased (Figure 5A). The Western blot results revealed that the cleavage efficiency of Caspase3 was reduced significantly in CXCL8-knockout cells that were treated with PMT at a concentration of 10 μg/mL; CXCL8-knockout cells appear to show a cleavage of Caspase3 with PMT at a concentration of 30 μg/mL (Figure 5B). This finding indicates that the *CXCL8* gene mediates PMT-induced apoptosis. Confirming this result, a flow cytometry showed that the apoptosis rates of CXCL8-knockout cells were 6.55%, including 0.95% early apoptosis and 5.60% late apoptosis, and 7.21%, including 1.31% early apoptosis and 5.90% late apoptosis, which were significantly lower than the rates of normal PK15 cells—16.46%, including 0.46% early apoptosis and 16.00% late apoptosis, and 16.79%, including 0.59% early apoptosis and 16.20% late apoptosis—under the stimulation of PMT toxin at concentrations of 10 and 30 μg/mL (Figure 5C).

**Figure 6:**
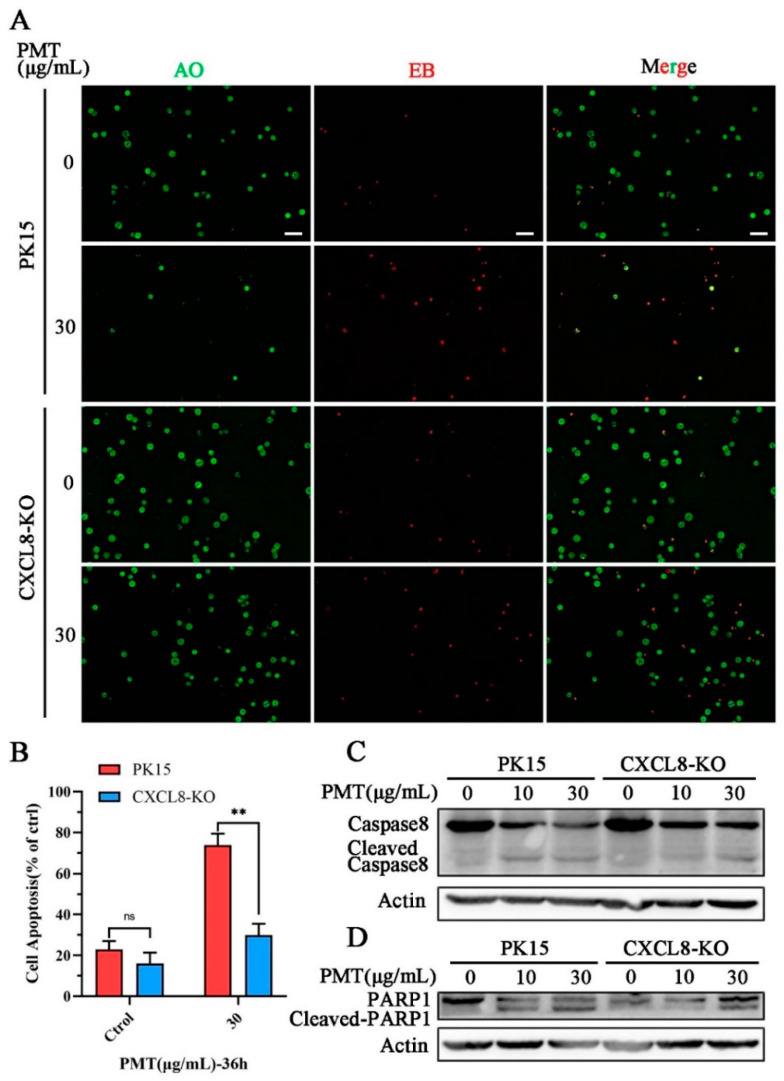
CXCL8 regulates PMT-induced apoptosis through Caspase8 pathway. (**A**) AO-EB detection of apoptosis in PK15 and CXCL8-KO cells after treatment with PMT (36 h, 37 °C). AO, normal cells (green); EB, apoptotic cells (red). Scale bar, 100 μm. (**B**) Quantification of tdata presented in (**A**). (**C**) Immunoblot analysis of Caspase-8-cleaved protein extracts from PK15 normal cells and CXCL8-KO cells after treatment with PMT (36 h, 37 °C). (**D**) Immunoblot analysis of PARP1-cleaved protein extracts from PK15 normal cells and CXCL8-KO cells after treatment with PMT (36 h, 37 °C). Data are represented as means ± S.D.; *n* = 3. ns > 0.05, ** *p* < 0.01.

Furthermore, AO-EB staining revealed that CXCL8-knockout cells resisted PMT-induced apoptosis (Figure 6A,B). We detected the expression of the exogenous apoptotic pathway Caspase 8 protein, compared with CXCL8-knockout cells, and found that PMT promoted the cleavage of exogenous apoptotic Caspase 8 at a concentration of 10 μg/mL (Figure 6C). In addition, we confirmed that PMT induces activation of PARP1 and found that PMT promotes the cleavage of PARP1 between WT cells and CXCL8-knockout cells (Figure 6D). Compared to PK15 cells, the cleavage of Caspase8 and PARP1 were inhibited with PMT at a concentration of 10 μg/mL in CXCL8-knockout cells. It indicates that CXCL8 loss results the resistance of PK15 cells to PMT-induced exogenous apoptosis.

**Figure 7:**
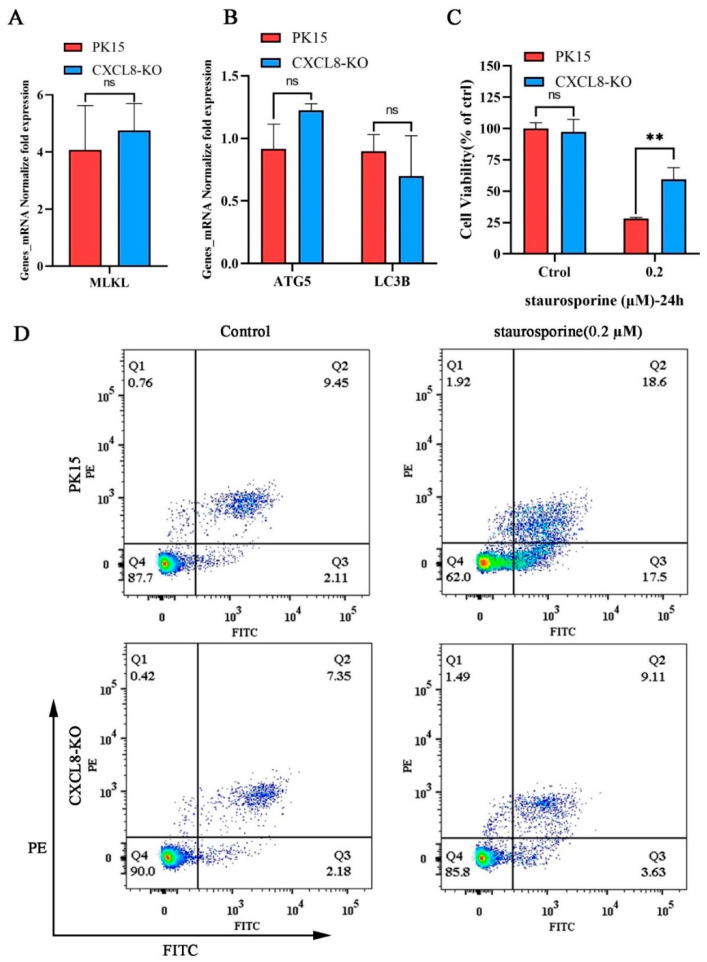
CXCL8 resisted staurosporine-induced cell apoptosis in PK15 cells. (**A**) mRNA level of necroptosis-related gene *MLKL* in PK15 and CXCL8-KO cells was detected using qRT-PCR after incubation with PMT. (**B**) mRNA level of autophagy-related gene *ATG5* and *LC3B* in PK15 and CXCL8-KO cells was detected using qRT-PCR after incubation with PMT. (**C**) Cell viability assay of PK15 and CXCL8-KO cells by CCK-8 after incubation with staurosporine (0.2 μM). Data are represented as means ± S.D.; *n* = 3. ns > 0.05, ** *p* < 0.01. (**D**) Flow cytometry detection of apoptosis rate in PK15 and CXCL8-KO cells under staurosporine (0.2 μM) stimulation. Q1, the percentage of necrotic cells; Q2, late the percentage of apoptotic cell; Q3, early the percentage of apoptotic cell; Q4, the percentage of normal cells.

At the same time, there was no significant difference in the transcription levels of the autophagy-related gene *ATG5*, *LC3B*, and the necroptosis marker gene *MLKL* after CXCL8-knockout cells were stimulated by PMT (Figure 7A,B). In addition, we treated PK15 and CXCL8-KO cells with staurosporine as an apoptosis inducer. Compared to PK15 cells, CXCL8-KO can significantly resist cell death caused by staurosporine (Figure 7C and Figure S4). Subsequently, flow cytometry observation revealed that CXCL8-knockout cells significantly resist apoptosis caused by staurosporine; the apoptosis rate of CXCL8-knockout cells was 12.74%, including 3.63% early apoptosis and 9.11% late apoptosis, which were significantly lower than the rates of normal PK15 cells—36.10% including 17.50% early apoptosis and 18.60% late apoptosis—under the stimulation of staurosporine at concentrations of 0.2 μM (Figure 7D). This finding indicates that CXCL8-KO cells resisted PMT-induced cell death mainly by inhibiting the apoptotic pathway. The knockdown of CXCL8 reduced PMT-induced apoptosis in PK15 cells, suggesting that CXCL8 plays a key role in the PMT-induced cell death pathway.

## 3. Discussion

Pathogenic bacteria infect hosts by employing virulence factors. PMT, the main virulence factor of *P. multocida*, causes swine atrophic rhinitis and pneumonia, which affects the healthy development of the pig-breeding industry [53]. However, there is still little research on which host factors are involved in the pathogenic mechanism of PMT. We used porcine kidney-15 (PK15) cells as a model to explore the cytotoxic effect of PMT and found that PMT can cause PK15 cell death. To further explore the host gene changes induced by PMT in PK15 cells, a transcriptome sequencing analysis found that *CXCL8* was significantly upregulated under PMT treatment. When the *CXCL8* gene was “knocked out”, it increased cell viability under PMT treatment. This suggests that CXCL8 plays a key role in the PMT-induced death of PK15 cells.

In order to study PMT-induced cytotoxicity, we used prokaryotic recombination technology to express and prepare recombinant PMT. Because of the existence of endotoxin, it not only affects the growth of cells but also causes damage to the animal body, which can cause a severe inflammatory response, thereby affecting the accuracy of the test [54,55]. After ultrafiltration and endotoxin removal, a recombinant PMT protein with high purity was obtained to avoid the influence of endotoxin and other impurities on the experimental results. Using the Triton-114 method [56], endotoxin was removed from the concentrated PMT protein. Figure S5 and Table S1 showed that the endotoxin content was 0.26 EU/mL. Most previous studies have focused on the transport of the PMT toxin protein, and the cell proliferation caused by it [15]. Previous studies have reported that PMT mainly caused Vero cell death and skin necrosis in guinea pigs, as a model for evaluating PMT. However, the relevant cytotoxicity of pigs, which is the primary host, has not been reported [57]. We explored the toxicity of PMT to other pig-derived cells, such as PAM, ST, and IPEC cells, which showed no significant cell death compared with PK15 cells (Figure S6). It was reported that a PMT mutant without catalytic activity (PMT-C1165S) was used as a control to study the toxicity of PMT [58,59]. To further confirm that PMT causes PK15 cell death, we determined that the PMT inactivating protein PMT-C1165S has no cytotoxicity to PK15 (Figure 1E). In addition, to exclude the effect of endotoxin on cytotoxicity, PK15 cell death cannot be induced under the stimulation of LPS at different concentrations (Figure S7). To reveal early sensitive changes in gene levels after PMT treatment, we measured and analyzed the differential changes in gene transcription levels in PK15 cells induced after 24 h of PMT-exposure treatment. We found that after PMT treatment, the transcription levels of partial genes were upregulated. Cytokines, such as IFNs and chemokines, play pivotal roles in inflammatory responses and anti-tumor immunity [60]. Many of the top 12 upregulated genes encode inflammatory cytokines and proteins, such as IRF1, OAS2, MX2, IFIT1, and ISG15, which are involved in the interferon signal pathway [61]. While other genes such as *LAMA3*, *DUSP5*, *CSF2*, and *ZBP1* play an important role in tumor immune signaling pathways, these genes not only play an important role in anti-tumor activity but also participate in the cell death pathway [62]. It is reported [63] that the secretion regulation of CXCL8 also participates in the interferon signal pathway, and CXCL8 is expressed in numerous cells involved in the formation of tumors. It has been reported that IFN-γ promotes PANoptosis in PMT-induced pneumonia in mice [64]. This indicates that inflammatory factors are crucial in PMT toxicity. Under PMT stimulation, *CXCL8* gene transcription was significantly induced, with the highest fold change, consistent with the transcriptome results. In addition, under PMT-C1165S and LPS stimulation, *CXCL8* gene transcription was not considerably induced (Figure S8). This result documents that CXCL8 plays a vital role in PMT-induced PK15 cell death. PMT causes PK15 cell death and stimulates the transcription of the *CXCL8* gene to be significantly upregulated. The upregulation of CXCL8 expression promotes the expression of anti-apoptotic genes, and then promotes the occurrence and metastasis of tumors [37,39], which may indicate that CXCL8 plays a negative-feedback regulation role in mediating PK15 cell death; relevant in-depth research should be rewarding.

According to the transcriptome sequencing results, some genes involved in cell death rank high. We first detected the apoptosis pathway and found that PMT can stimulate apoptosis. CRISPR/Cas9 gene-editing technology has been widely used for gene knockout [65]. We used this technology to knock out the *CXCL8* gene, and the expression of CXCL8 could no longer be detected by Western blot, likely because the antibody from human CXCL8 could not recognize the porcine CXCL8 protein. In addition, we detected the expression level of CXCL8 in knockout cells with the swine CXCL8 ELISA kit. We found that the intracellular CXCL8 expression level was significantly reduced in knockout cells, while the extracellular CXCL8 expression level could not be detected in PK15 and CXCL8-KO cells, indicating that IL8 plays a role in regulating intracellular signaling pathways. Two sgRNAs were designed to target the *CXCL8* gene to exclude off-target effects. It showed that CXCL8-knockout cells could significantly resist PMT-induced cell death. We conducted a screening of monoclonal CXCL8-KO cell lines by the limited dilution method. Using DNA sequencing to find a frameshift mutation at the target site of CXCL8 in knockout cells (Figure S9), we found that the transcriptional level of CXCL8 in knockout cells was significantly reduced. We predicted the potential off-target sites of CXCL8 through the CHOPCHOP website; Sanger sequencing demonstrated that four potential off-target sites showed no off-target effects (Figure S10 and Table S2), thus proving that the *CXCL8* gene was knocked out. It has been reported that PMT can stimulate multiple signaling pathways in cells [66]. Also, CXCL8 only affects one of the pathways and reduces the cytotoxicity of PMT. CXCL8 is well-known for its operation through pro-inflammatory signaling pathways such as the NF-κB and MAPK pathways [41,66], which are known to play roles in cell survival and apoptosis. It is also known from the literature that CXCL8 can promote survival signals like PI3K/AKT signaling [67], thereby promoting cell survival and anti-apoptotic effects. CXCL8-KO cells resist apoptosis caused by PMT, which may be the reason for differences in different cell lines. According to the transcriptome data, after PMT exposure, the exon content of transcriptome genes decreases, while the intron content increases (Figure S11). It can be inferred that PMT exposure affects the splicing of the host gene mRNA and increases the mRNA transcripts of its genes. We speculate that PMT toxin promotes the endogenous transcription of CXCL8, thereby promoting the expression of CXCL8. We also assume that there are specific differences in the intracellular and extracellular stimulation pathways of CXCL8. This leads to CXCL8-deficient cells resisting apoptosis, and the specific signaling pathway is worth further research.

Our further detection and analysis revealed that, after the *CXCL8* gene was knocked out, the transcription level of the apoptosis marker Caspase3 decreased under the stimulation of PMT at a concentration of 10 μg/mL, and the cleavage efficiency of Caspase3 was decreased; this result indicated that CXCL8 affected PMT-induced PK15 apoptosis. At the same time, the apoptosis rate of CXCL8-knockout cells was significantly lower than that of PK15 normal cells under PMT stimulation. Moreover, after *CXCL8* gene knockout, cell apoptosis was significantly inhibited, and there were significantly fewer dead cells under PMT stimulation, according to the AO-EB assay. Caspase8 and PARP1 were cleaved, leading to the activation of the endogenous apoptosis pathway. As for cell necrosis and autophagy-related marker genes *MLKL*, *ATG5*, and *LC3B* [68,69], there was no significant difference in the transcription levels of CXCL8 knockout under PMT stimulation. Furthermore, we speculate that the resistance of CXCL8 deficiency to cell apoptosis may not be direct but indirect. It is possibly its effects on the cellular microenvironment or some obscure signaling pathways that generally impact cell survival versus death. As we speculated, CXCL8-knockout cells can significantly resist staurosporine-induced apoptosis, based on our research results, although this study only demonstrates the role of IL8 through apoptosis; the specifically related pathways and downstream proteins have not been thoroughly studied, which will be the direction for further in-depth research. In a word, the discovery of CXCL8, a key cytokine that can resist apoptosis, provides ideas for studying apoptosis pathways and a target basis for exploring how to develop drugs resistant to the *P. multocida* toxin from the host perspective.

## 4. Material and Methods

### 4.1. Cell lines, Bacterial Strains, Culture Conditions, and Antibodies

PK15 cells were cultured in Dulbecco’s Modified Eagle Medium (DMEM; Gibco, Carlsbad, CA, USA), supplemented with 0.37% sodium bicarbonate, 100 U/mL penicillin-streptomycin (Solarbio, Beijing, China), and 10% fetal bovine serum (FBS; PNA, Adenbach, Germany) in a humidified atmosphere of 95% air and 5% CO_2_ at 37 °C. The *Pasteurella multocida* strain HN06 was donated by Huazhong Agricultural University [70] and grown in tryptic soy broth Difco or tryptic soy agar supplemented with 5% fetal calf serum (Gibco, Carlsbad, CA, USA). *E. coli* strains were grown in liquid Luria-Bertani medium or agar Difco. Active and pro-Caspase-3 rabbit monoclonal antibody (A19654) and anti-ACTB rabbit polyclonal antibody (AC026) were obtained from Abclonal (Wuhan, China). Rabbit polyclonal antibody anti-Caspase 8/p43/p18 (13423-1-AP) and anti-PARP1 rabbit Polyclonal antibody were purchased from Proteintech (Chicago, IL, USA).

### 4.2. Cloning, Expression, and Purification of PMT or PMT-C1165S Recombinant Protein

Construction of the vectors pCold I-ToxA: The *ToxA* gene expressing the PMT protein was amplified from *P. multocida* strain HN06 using the primers sequences F: accctcgagggatccgaattcATGAAAACAAAACATTTTTTTAACTCAG; R: agcagagattacctatctagaTTATAGTGCTCTTGTTAAGCGAGGC. The gene was ligated into pCold I vector with a seamless mix (Biomed, Beijing, China). Sequencing confirmed the recombinant plasmid of pCold I-ToxA (Sangon Biotech, Shanghai, China). The construction of the point mutation vectors pCold I-PMT-C1165S: PMT-C1165S was amplified from pCold I-ToxA using the primers sequences F: TGGAAGCTGGCTCTTCTGATTCAGTA; R GAAGAGCCAGCTTCCATACACACTAA. Sequencing confirmed the recombinant plasmid of pCold I-PMT-C1165S (Sangon Biotech, Shanghai, China). The expression of PMT and PMT-C1165S: pCold I-PMT-C1165S and pCold I-ToxA were transformed into *E. coli* BL21 (DE3)-competent cells (Cowin Biotech, Chengdu, China). A total of 1 mM isopropyl-beta-D-1-thiogalactopyranoside was added to induce expression for 24 h. The cells were collected for sonication. The supernatant was collected by centrifugation and added to a standard protein-loading buffer (5×) (Beyotime, Shanghai, China) and bathed in boiling water for 10 min. The expression of the PMT protein was detected with SDS-PAGE. The supernatant from the sonication was purified with Ni2+ affinity chromatography, using Profinity™ IMAC Ni-Charged Resin (Bio-Rad, Hercules, CA, USA). The concentration of PMT was determined with a BCA protein assay kit (Biomed, Marina del Rey, CA, USA).

### 4.3. Removal and Detection of Endotoxin for PMT or PMT-C1165S Recombinant Protein

The purified PMT or PMT-C1165S protein was added to 1% Triton X-114, stirred with magnetic force for 60 min at 4 °C, and placed in a constant-temperature water bath at 30 °C for 40 min, and the mixture was centrifuged at 15,000× *g* for 15 min at 25 °C. The separated upper water phase is the PMT protein with the removal of endotoxin. The PMT protein was dialyzed with a dialysis bag and concentrated with a 50 kDa ultrafiltration tube (Millipore, Burlington, MA, USA). Then, the endotoxin content standard curve was established according to the instruction manual, and the endotoxin content in the PMT toxin protein sample was determined using the endotoxin test kit (Bioendo, Xiamen, China). The concentration of PMT was determined with a BCA protein assay kit (Biomed, Beijing, China). The sample was stored in a refrigerator at −80 °C.

### 4.4. PMT and PMT-C1165S Toxin in the Treatment of Cells

PK15 cells with good growth were inoculated in 6-well plates at 25,000 cells/mL. When the cell confluence reached 90%, the old medium was removed, and the cells were washed with PBS. PMT and PMT-C1165S diluted with serum-free DMEM were added to the test group at 10 μg/mL and 30 μg/mL. Normal PK15 cells without exposure were set as a blank control and placed in a constant-temperature incubator (containing 5% CO_2_) at 37 °C for 24 h and 36 h and photographed under an inverted microscope.

### 4.5. Sample Preparation and RNA Extraction

PK15 cells were seeded in T75 cell culture flasks. When the cell confluence reached 90%, the cells were treated with PMT at a concentration of 30 μg/mL. Normal PK15 cells without exposure were set as a blank control. Two flasks of cells from each group were used as two independent biological replicates, for a total of four samples. After 24 h, cell samples were collected. Total RNA was extracted with a Trizol reagent kit (Invitrogen, Carlsbad, CA, USA). RNA quality was assessed on an Agilent 2100 Bioanalyzer (Agilent Technologies, Palo Alto, CA, USA) and checked with RNase-free agarose gel electrophoresis.

### 4.6. RNA Sequencing and Analysis of Samples

The enriched mRNA was fragmented into short fragments with fragmentation buffer and reversely transcribed into cDNA with the NEBNext Ultra RNA Library Prep Kit for Illumina (NEB#7530, New England Biolabs, Ipswich, MA, USA). Purified double-stranded cDNA fragments were end-repaired. A base was added and ligated to Illumina sequencing adapters Illumina Novaseq6000 by Gene Denovo Biotechnology Co (Guangzhou, China). The ligation reaction was purified with AMPure XP Beads (1.0×). Ligated fragments were size-selected by agarose gel electrophoresis and amplified by polymerase chain reaction (PCR). The resultant cDNA library was sequenced using Illumina.

To get high-quality clean reads, reads were further filtered by Fastp (version 0.18.0). The short reads alignment tool Bowtie2 (version 2.2.8) was used for mapping reads to the ribosome RNA (rRNA) database. The mapped reads of each sample were assembled with StringTie v1.3.1 in a reference-based approach. An FPKM (fragment per kilobase of transcript per million mapped reads) value for each transcript region was calculated, to quantify its expression abundance and variations, using RSEM software (version 1.3.3). A principal component analysis was performed with R package g models (http://www.rproject.org/ (accessed on 30 July 2021)). An RNA differential expression analysis was performed with DESeq2 software (version 1.10) between two different groups (and by edgeR between two samples). Genes/transcripts with the criteria of a false discovery rate less than 0.05 and absolute fold change ≥ 2 were considered differentially expressed genes/transcripts.

### 4.7. Quantitative RT-PCR (qRT-PCR)

Total RNA was extracted from PMT-treated cells with a UNIQ-10 column TRIzol Total RNA Isolation Kit (Sangon, Shanghai, China) and subjected to reverse transcription with the PrimeScript RT Reagent Kit with gDNA Eraser (Takara, Beijing, China). The cDNA templates were used for qRT-PCR with TB Green Premix Ex Taq II (Takara, China) and the primers listed in Table 3. The relative mRNA levels of the genes were calculated with the 2^−ΔΔCT^ method, with the porcine *GAPDH* gene as a control. The qRT-PCR conditions were 95 °C for 30 s, followed by 40 cycles of 95 °C for 5 s, 60 °C for 30 s, and 72 °C for 30 s, using the LightCycler 96 system (Roche, Basel, Switzerland). Each experiment consisted of three biological replicates, and qRT-PCR for each sample was performed in triplicate.

### 4.8. Generation of CXCL8-Knockout Cells Using the CRISPR/Cas9 System

The sgRNA targeting the *CXCL8* gene in PK15 cells was designed, synthesized, and inserted into the vector lentiCRISPR v2 (Addgene plasmid 52961) to generate deletion or insertion mutations. The sgRNA sequences were sgRNA1-F, 5′-3′ CACCGTAAGCTTGTCAATGGAAAAG; sgRNA1-R, 5′-3′ AAACCTTTTCCATTGACAAGCTTAC. and sgRNA2-F, 5′-3′ CACCGTTCCTTGATAAATTTGGGG; sgRNA2-R, 5′-3′ CAAACCCCAAATTTATCAAGGAAC. The constructed lentiviral vector plasmid and psPAX2 (Addgene plasmid 12260) and pMD2.G (Addgene plasmid 12259) were transfected into 293T cells at a ratio of 5:3:2. Cell supernatants were collecte 48 h after transfection and centrifuged at 300× *g* at 4 °C for 5 min. When the confluency of PK15 cells reached about 50%, the lentiviral solution was added for 48 h, and 7 μg/mL of puromycin (Solarbio, China) was added for resistance screening for 7 d. Cells were collected for the extraction of DNA, amplified, and sequenced with knockout identification primers. The primer sequences were KO1-F, AAATTGGCTTGGGCTTAG; KO1-R, CGCTTGCCCATACTTTCT. KO2-F, TTCTGGCAAGAGTAAGTGCAGA; KO2-R, TTTAAATGCCATGCAGACAAAG. The knockout efficiency of the *CXCL8* gene-knockout cell lines was detected with qRT-PCR.

### 4.9. CXCL8 ELISA Assay

PK15 cells and CXCL8-knockout cells with good growth were inoculated in 6-well plates at 25,000 cells/mL. When the cell confluence reached 90%, the old medium was collected, and the cells were washed with PBS. Then, they were incubated with RIPA lysis buffer containing the protease inhibitor PMSF (Solarbio, Beijing, China) for 15 min at 4 °C. Lysates were centrifuged at 13,400× *g* for 5 min at 4 °C. The supernatants were collected, and the expression of CXCL8 in the old medium and the supernatants were detected by the swine CXCL8 ELISA kit (BYabscience, Nanjing, China).

### 4.10. Cell Viability Assay

The cell density was adjusted to 5000 cells/150 μL. PK15 cells or a CXCL8-knockout cell suspension were spread in 96-well plates at 150 μL/well and cultured in a constant-temperature incubator (containing 5% CO_2_) at 37 °C. When the adherent growth reached a 90% confluence, the old medium was removed, and the cells were washed once with PBS. Staurosporine (0.2 μM) or PMT protein diluted with serum-free DMEM at a concentration of 10 and 30 μg/mL was added to the cells of each experimental group. Mock-treated cells and DMEM without PMT or staurosporine served as the negative control and blank control, respectively. The cells were kept at 37 °C, and then cultured in a constant-temperature incubator (containing 5% CO_2_) with PMT for 36 h or with staurosporine for 24 h. A total of 15 µL of CCK-8 reagent (Meilun, Dalian, China) was added to each well, and the cells were incubated for 1 h at 37 °C. Absorbance was measured at a wavelength of 450 nm with a microplate reader (Thermo Scientific, Waltham, MA, USA). Cell viability was calculated as [(mean OD450 treatment − mean OD450 blank)/(mean OD450 control − mean OD450 blank)] × 100%.

### 4.11. LDH Release Assay

The cytotoxicity of PMT was assessed with the LDH cytotoxicity assay kit (LDH) (Biomed, China). The operation steps were like those of the cytotoxicity detection method. The cells without PMT were set as the positive group. After 36 h of PMT exposure, 15 μL/well of LDH release reagent was added to the cells in the positive group, and incubation was continued in the cell incubator after repeated pipetting and mixing. After 1 h, the 96-well plate was centrifuged at 400× *g* for 5 min, and the supernatant of about 120 µL was transferred to a new 96-well plate. Sixty μL of LDH detection working solution was added to each well. The cells were mixed well, and then incubated in the dark at room temperature for 30 min. Absorbance was measured at a wavelength of 490 nm with a microplate reader (Thermo Scientific, Waltham, MA, USA). Cytotoxicity was calculated as [(mean OD490 treatment − mean OD490 blank)/(mean OD490 positive − mean OD490 blank)] × 100%.

### 4.12. Western Blotting Analysis

PMT was diluted by DMEM without serum. PK15 and CXCL8-knockout cells were harvested after PMT treatment for 36 h. The cells were washed twice with cold PBS, then incubated with RIPA lysis buffer containing the protease inhibitor PMSF (Solarbio, Beijing, China) for 15 min at 4 °C. Lysates were centrifuged at 13,400× *g* for 5 min at 4 °C. The supernatants were collected and mixed with 5 × protein-loading buffer and boiled in water for 10 min. Ten μL was loaded for SDS-PAGE electrophoresis at 80 V for 20 min, then 120 V for 60 min. The transfer of protein samples from the gel to the PVDF membrane was performed by the application of an electrical current (200 mA for 60 min). After the transfer, samples were placed in a blocking solution of 5% *w*/*v* skim milk in TBST [0.05% Tween 20, 0.15 M NaCl, and 1 M Tris-HCL, pH 7.5], incubated at room temperature for 1.5 h, and incubated overnight with primary antibodies diluted in primary antibody dilution buffer (Beyotime, China). Membranes were washed 4 times for 5 min each with TBST. Then, 5000-fold diluted HRP-conjugated goat anti-rabbit or anti-mouse IgG was added to the membranes and incubated at room temperature for 60 min on a shaker. The membranes were washed 4 times for 5 min each with TBST, then developed by adding ECL reagents (Bio-Rad, Hercules, CA, USA) according to the manufacturer’s instructions.

### 4.13. Flow Cytometry

Apoptosis was evaluated by flow cytometry. PK15 and CXCL8-knockout cells were stimulated with PMT at concentrations of 10 and 30 μg/mL for 36 h or with staurosporine at concentrations of 0.2 μM for 24 h. The cells were separated, centrifuged, and counted, and 1 × 10^6^ cells were taken. After washing with PBS and centrifuging, 200 µL 1× buffer (0.1 M Hepes pH 7.4, 1.4 M NaCl, and 25 mM CaCl_2_ solution) was added to the collected cells, and 2 μL Annexin V/PE and 4 μL FITC solution was added (Becton, Dickinson and Company, New York, NY, USA). The cells were incubated in the dark at room temperature for 15 min, 300 μL PBS was added to the EP tube, and the apoptosis rate was measured by flow cytometry (Thermo Scientific, Waltham, MA, USA).

### 4.14. AO-EB Apoptosis Assay

The PK15 and CXCL8-knockout cells were plated in a 12-well plate. When the cell density reached 90%, the original medium was removed, and the cells were washed with PBS. PMT diluted with serum-free DMEM at a concentration of 30 μg/mL was added. The treatment group and control group were cultured in a constant-temperature incubator at 37 °C for 36 h. The medium was removed, the cells were washed twice with PBS, then digested with PBS containing 10% trypsin and centrifuged at 100× *g* for 5 min to obtain cell pellets. Then, 500 μL of the prepared AO-EB working solution (1 mL of PBS plus 10 μL of AO (acridine) solution and 10 μL of EB (ethidium bromide) solution) was added to each sample. After being at room temperature for 10 min, 10 μL of the suspension was dropped onto a glass slide and covered with a coverslip. The cells were observed and photographed under an inverted fluorescence microscope.

### 4.15. Statistical Analysis

Quantitative data are presented as the means ± standard deviations (SD). Student’s *t*-test was used to measure significant differences between the two groups with GraphPad Prism 9 software (GraphPad Software, Inc., San Diego, CA, USA). Differences were considered statistically significant if the *p*-value was <0.05.

## 5. Conclusions

In this study of the mechanism of *Pasteurella multocida* toxin (PMT) cytotoxicity, transcriptome sequencing analysis revealed that CXCL8 is the critical host factor; the knockout of CXCL8 in PK15 cells by CRISPR/Cas9 helped to resist PMT-induced PK15 cell death, and it decreased the cleavage of Caspase3, Caspase8, and PARP1 in the apoptosis pathway, thereby resisting the apoptosis of PK15 cells induced by PMT. We documented that CXCL8 mediates PMT-induced PK15 cell death through the apoptotic pathway. This new information may be useful in the development of measures to prevent the severe consequences of *Pasteurella multocida* infection and provide a basis for using PMT or CXCL8 to modulate the host immune response.

## Figures and Tables

**Table 1:** Summary statistics for quality control of sequencing data.

Sample	Raw Reads	Clean Reads (%)	Raw Bases (G)	Clean Bases (G)	Q20 (%)	Q30 (%)	GC Content (%)
PK15-1	46,501,322	46,292,164 (99.55%)	6.98	6.91	98.17	94.75	48.33
PK15-2	54,439,480	54,200,760 (99.56%)	8.17	8.10	98.19	94.78	48.16
PK15-PMT-1	43,584,530	43,368,538 (99.50%)	6.54	6.48	98.07	94.52	47.24
PK15-PMT-2	49,633,044	49,401,324 (99.53%)	7.44	7.38	98.07	94.53	46.66

**Table 2:** Summary statistics of clean reads mapped to reference genome.

Sample	Total Reads	Unique Mapped Reads (%)	Total Mapped Reads (%)	Exon (%)	Intron (%)	Intergenic (%)	Expressed Genes	Novel Genes
PK15-1	46,151,464	89.83	95.22%	72.62	22.30	5.08	14819	908
PK15-2	54,026,874	90.11	95.43%	72.69	22.21	5.11	14932	913
PK15-PMT-1	42,926,818	88.31	94.82%	61.64	31.70	6.66	15073	903
PK15-PMT-2	48,861,884	88.13	94.50%	61.75	31.45	6.80	15249	916

**Table 3:** The primers used for qRT-PCR in this study.

Genes	Sequence (5′-3′)	For Construction of
*CXCL8*	F: AGCCCGTGTCAACATGACTT	RT-PCR for CXCL8 of PK15 cells
	R: TGGAAAGGTGTGGAATGCGT	
*LAMA3*	F: GAGGTGCTTGTGCAAACCTG	RT-PCR for LAMA3 of PK15 cells
	R: GTTCATGCAGTCTCCGGTCA	
*OAS2*	F: GATCCAACGGACCCAACCAA	RT-PCR for OAS2 of PK15 cells
	R: GCTTGGGGTCGAGTAGAGTG	
*IFIT1*	F: AAAGGCCAGAATGAGGGAGC	RT-PCR for IFIT1 of PK15 cells
	R: GCAAGCTTCCTGCAAGTGTC	
*MX2*	F: CATCGATCTTCCCGGCATCA	RT-PCR for MX2 of PK15 cells
	R: GTTGATGGTCTGCTGCTCCT	
*IRF1*	F: ATGGAAGGCCAACTTTCGCT	RT-PCR for IRF1 of PK15 cells
	R: CTGGTTCTTGGTGAGGGGTG	
*CSF2*	F: TGCCATCAAAGAAGCCCTGA	RT-PCR for CSF2 of PK15 cells
	R: GCTTGTACAGGTTCAGGCGA	
*RNF19B*	F: AATGCAGCGAGCGACTCAAC	RT-PCR for RNF19B of PK15 cells
	R: TTCGTACTTGTGCATGAGCG	
*ENDOV*	F: CCTCGGTGTCCTTACAGACG	RT-PCR for ENDOV of PK15 cells
	R: AGCCTTCAGGAGCCGTATCT	
*ISG15*	F: GGTGAGGAACGACAAGGGTC	RT-PCR for ISG15 of PK15 cells
	R: GGCTTGAGGTCATACTCCCC	
*DUSP5*	F: GCACGACCCACCTACACTAC	RT-PCR for DUSP5 of PK15 cells
	R: GCCCTTTTCCCTGACACAGT	
*ZBP1*	F: CAAGAGGCAGTTGCGATTCC	RT-PCR for ZBP1 of PK15 cells
	R: TTGACTTCCTTCGCCGTCTT	
*CCL5*	F: ACACCACACCCTGCTGTTTT	RT-PCR for CCL5 of PK15 cells
	R: TGTACTCCCGCACCCATTTC	
*CXCL2*	F: GAAGTTTGTCTCAACCCCGC	RT-PCR for CXCL2 of PK15 cells
	R: ATCAGTTGGCACTGCTCTTGT	
*TNFAIP3*	F: TGGAAACGGGGCTTTGCTAT	RT-PCR for TNFAIP3 of PK15 cells
	R: ATCTGTAGCATTCCTGGGCG	
*TNFSF9*	F: CCATCCTCAGGGAACTCAGC	RT-PCR for TNFSF9 of PK15 cells
	R: AGTCTTGGTCTAGGGGGTGG	
*IL11*	F: TGAACCTGACGCTTGACTGG	RT-PCR for IL11 of PK15 cells
	R: AACTGGCTTTGAAGGACGCT	
*IL1A*	F: ATGCCCGCAATCAAAGCATC	RT-PCR for IL1A of PK15 cells
	R: AACACGGGTTCGTCTTCGTT	
*IL18*	F: GCTGCTGAACCGGAAGACAA	RT-PCR for IL18 of PK15 cells
	R: AAACACGGCTTGATGTCCCT	
*Caspase3*	F: CCGAGGCACAGAATTGGACT	RT-PCR for Caspase3 of PK15 cells
	R: TCGCCAGGAATAGTAACCAGG	
*MLKL*	F: CGGGAAGGACGAAGGTATGGA	RT-PCR for MLKL of PK15 cells
	R: CGGTGGTTATCTCAGGGGAC	
*ATG5*	F: ACAGATGACAAAGATGTGCT	RT-PCR for ATG5 of PK15 cells
	R: CCTCCCGTTCAGTTATCTCA	
*LC3B*	F: CCGAACCTTCGAACAGAGAG	RT-PCR for LC3B of PK15 cells
	R: AGGCTTGGTTAGCATTGAGC	
*GAPDH*	F: ACATGGCCTCCAAGGAGTAAGA	RT-PCR for GAPDH of PK15 cells
	R: GATCGAGTTGGGGCTGTGACT	

## Data Availability

Data are contained within the article and Supplementary Materials.

## Supplementary Materials

The following supporting information can be downloaded at: https://www.mdpi.com/article/10.3390/ijms25105330/s1.
