# Supplementary material for: CXCL8 Knockout: A Key to Resisting Pasteurella multocida Toxin-Induced Cytotoxicity
Source: Int J Mol Sci. 2024 May 14;25(10):5330. doi: 10.3390/ijms25105330 (PMC11121343; doi:10.3390/ijms25105330)
Supplement: Supplementary file 1 [file ijms-25-05330-s001.zip › ijms-2963143-supplementary.pdf]

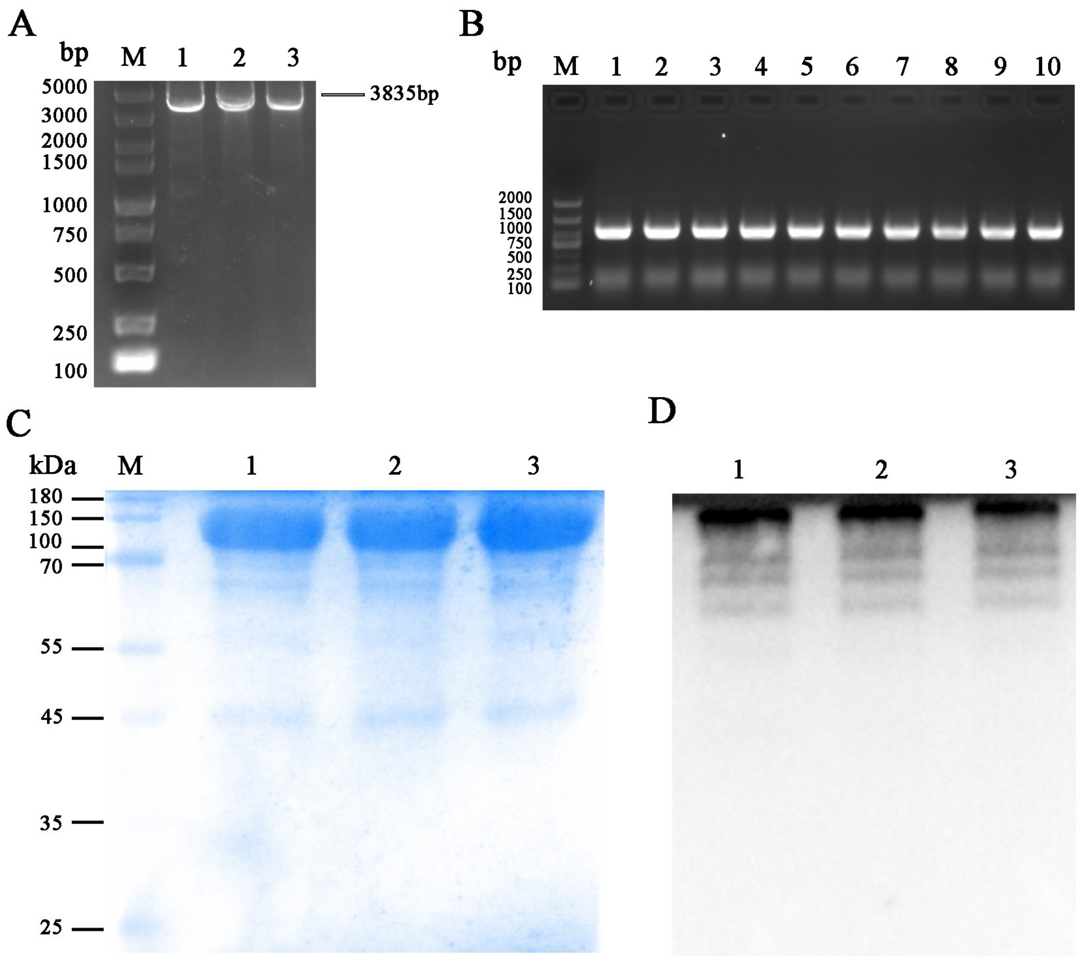

Figure S1. The expression and identification of PMT and PMT-C1165S

(A) The PCR amplification resμlts of ToxA gene. M, DL 5 000 DNA marker; 1-3, Amplification of ToxA gene. (B) Identification of recombinant plasmid pCold I-toxA. M, protein marker; 1-10: Identification of partial fragments of toxA gene. (C) The expression and purification of PMT-C1165S. M, protein marker; line 1-3, recombinant His-tagged PMT-C1165S. (D) Western-Blot identification of PMT-C1165S protein. Line 1-3, recombinant His-tagged PMT-C1165S.

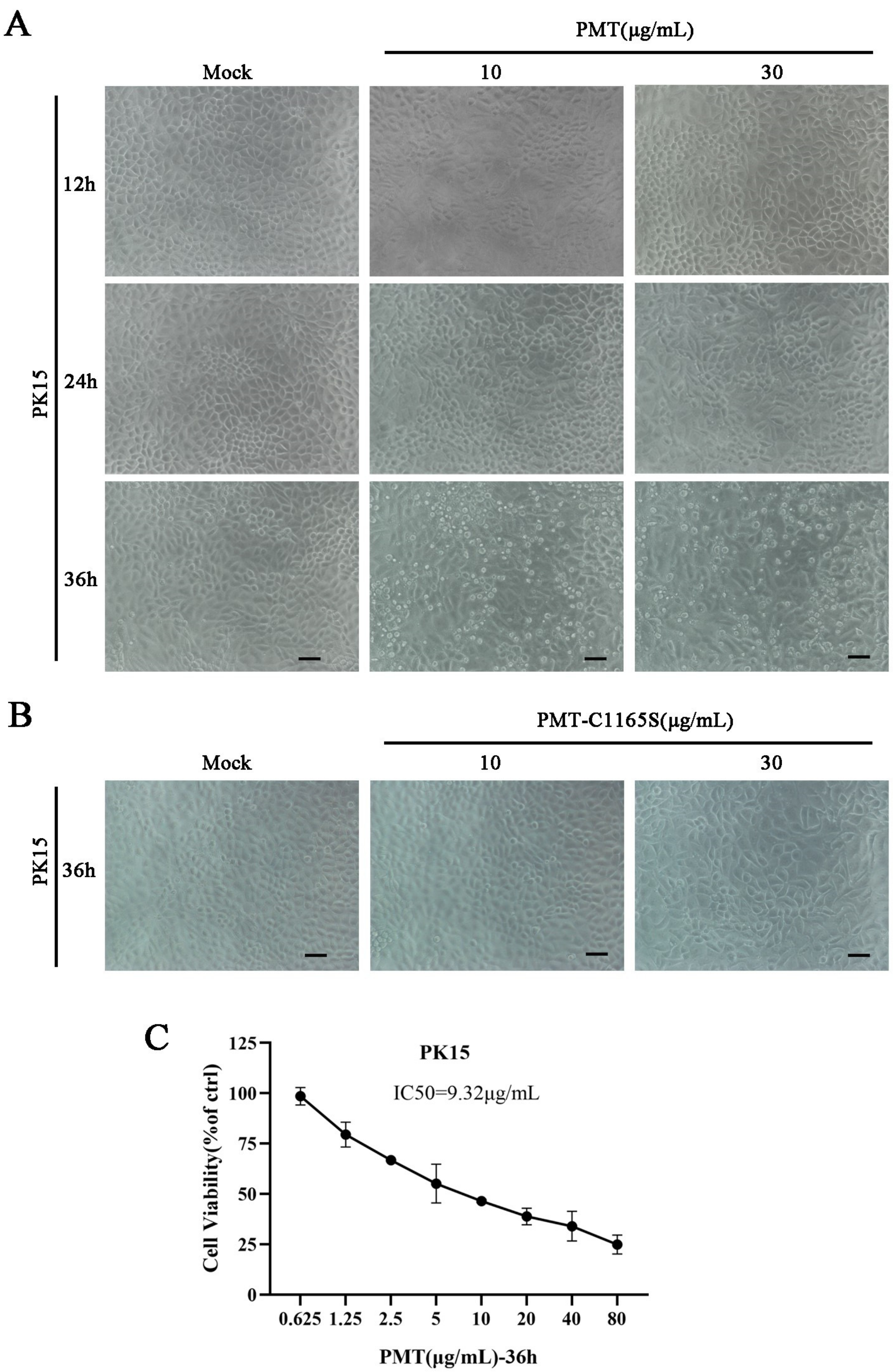

Figure S2. PMT and PMT-C1165S cytotoxicity on PK15 cells

(A) Cells were incubated with 10 and 30 μg/mL of PMT (12 h, 24 h, and 36 h, 37 °C)

and observed under a microscope. Scale bar, 100 μm. (B) Cells were incubated with 10 and 30 μg/mL of PMT-C1165S (36 h, 37 °C) and observed under a microscope. Scale bar, 100 μm. (C) Determination of PMT IC50 on PK15 cells.

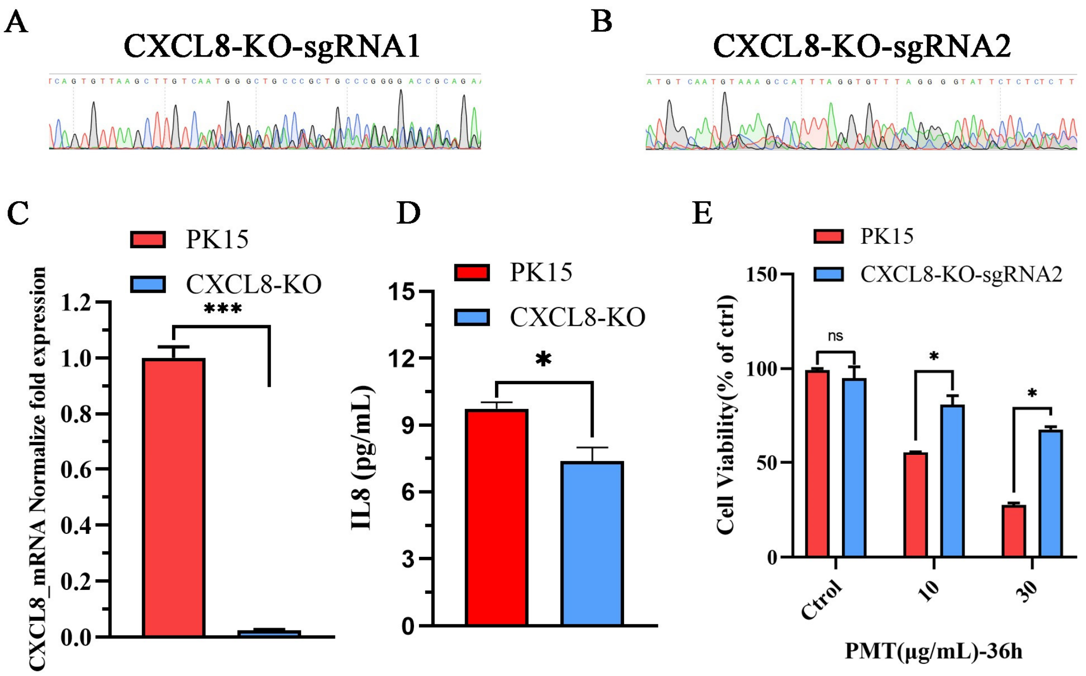

Figure S3. Identification of CXCL8 knockout cells

(A) Sequencing results of CXCL8 knockout cells from sgRNA1 targeting CXCL8 gene. (B) Sequencing results of CXCL8 knockout cells from sgRNA2 targeting CXCL8 gene. (C) Detection of CXCL8 gene transcription level using qRT-PCR on knockout cells. (D) Detection of CXCL8 expression level using swine CXCL8 ELISA assay kit on CXCL8-KO cells (E) Cell viability assay of PK15 and CXCL8-KO-sgRNA2 cells by CCK-8 after incubation with PMT. Data are represented as means ± S.D.; n = 3. * $P < 0.05$; ** $P < 0.01$; *** $P < 0.001$.

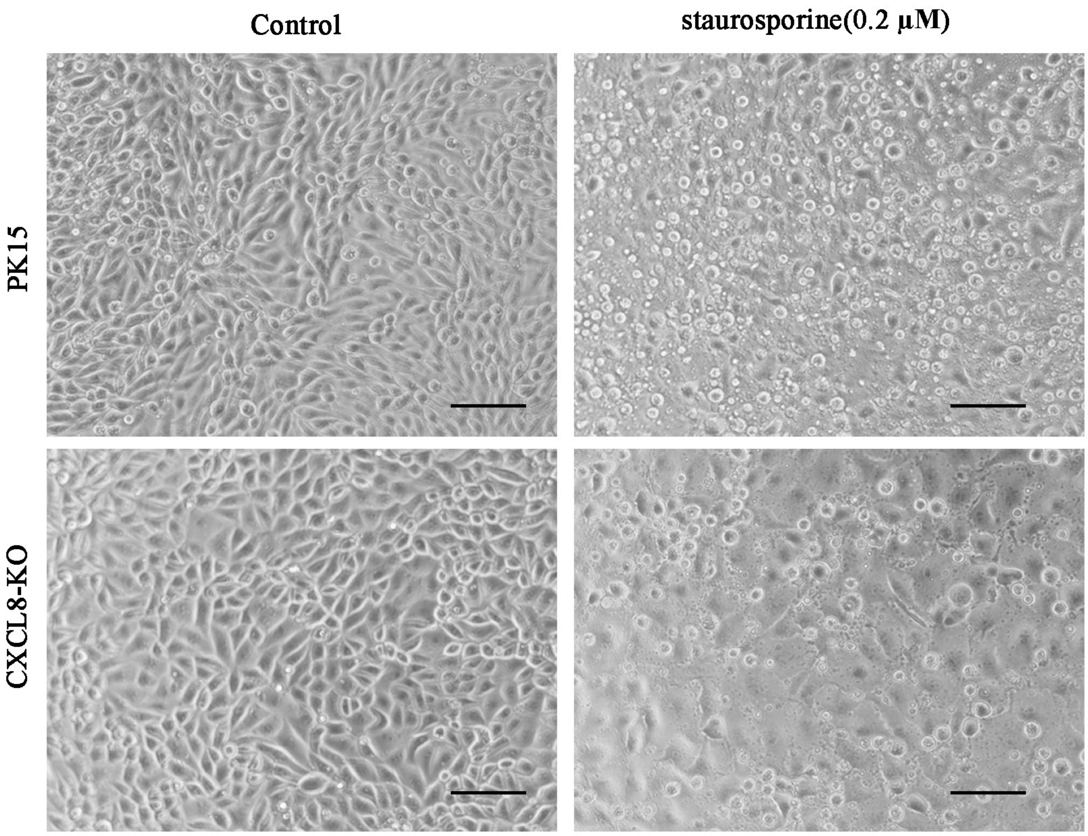

Figure S4. Staurosporine cytotoxicity on PK15 cells and CXCL8-KO cells. Cells were incubated with 0.2 μM of staurosporine (24 h, 37 ℃) and observed under a microscope. Scale bar, 100 μm.

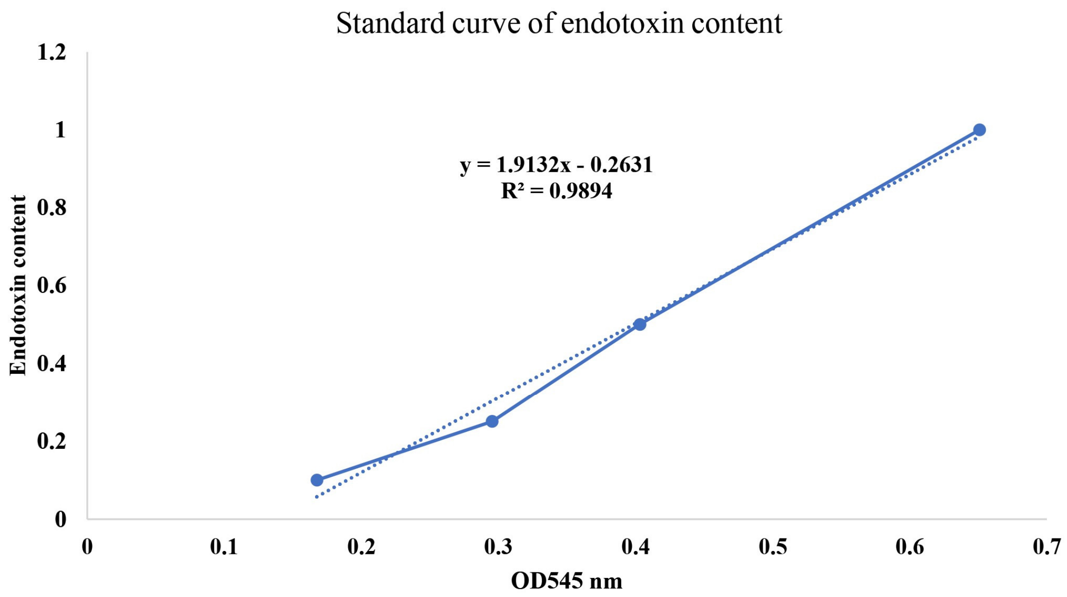

Figure S5. Standard curve of endotoxin content

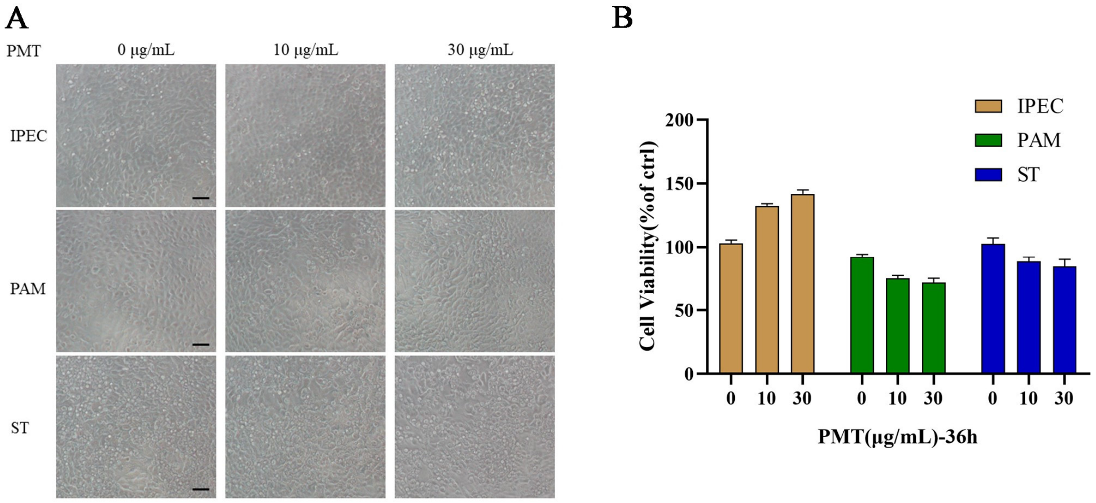

Figure S6. The toxicity of IPEC, PAM, and ST cells to PMT

(A) PMT's cytotoxicity on the IPEC, PAM, and ST cells. Cells were incubated with indicated concentrations of PMT (36 h, 37°C) and observed under microscope. Scale bar, 100 μm. (B) Cell viability assay of IPEC, PAM, and ST cells by CCK-8 after incubation with PMT.

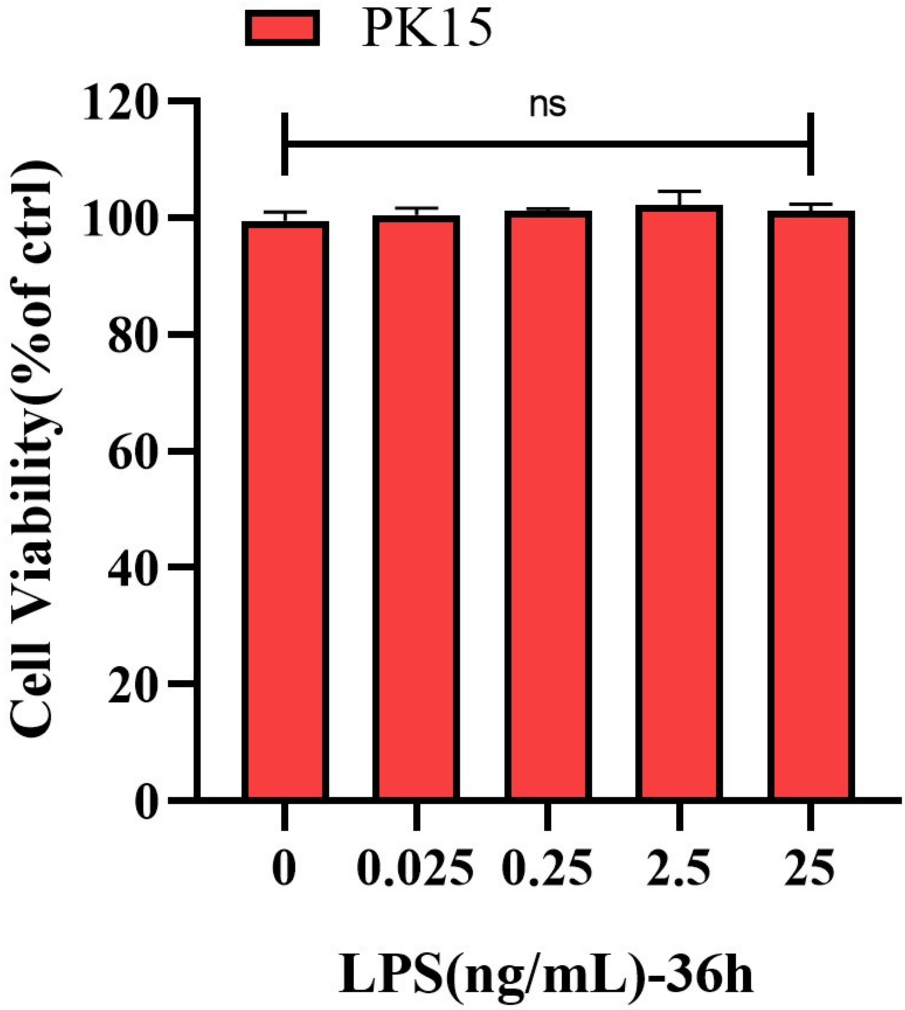

Figure S7. Toxicity of PK15 cells to LPS

LPS cytotoxicity on the PK15 cells. Cell viability assay of PK15 cells by CCK-8 after incubation with LPS.

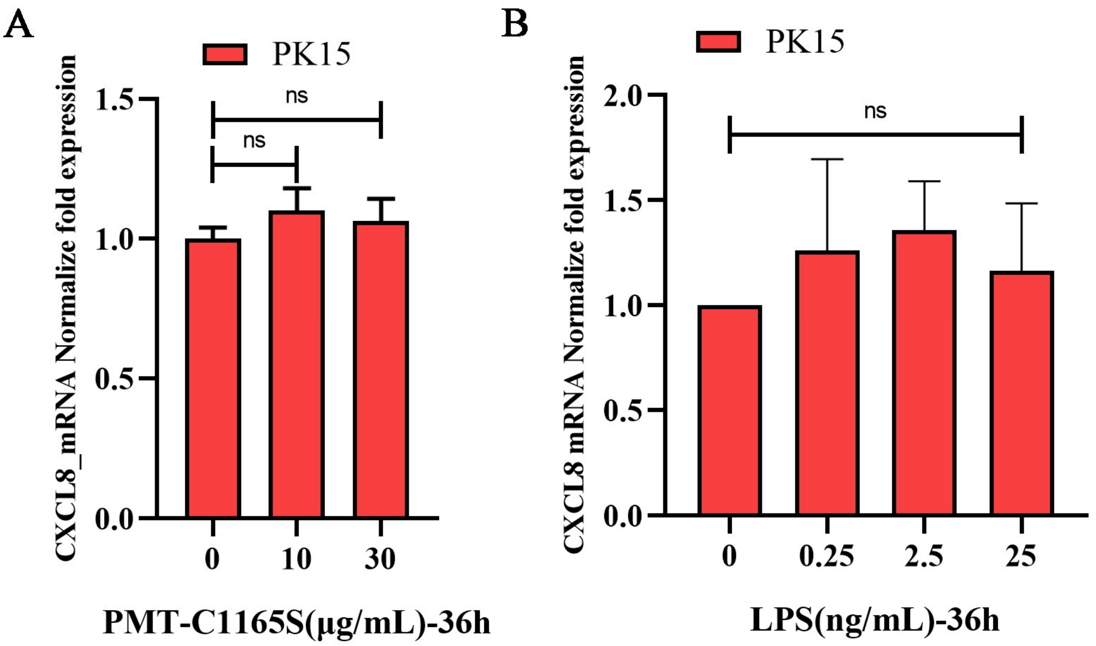

Figure S8. The transcription levels of CXCL8 by PMT-C1165S and LPS treated in the PK15 cells

(A) The mRNA level of CXCL8 in PK15 cells was detected using qRT-PCR after incubation with PMT-C1165S. (B) The mRNA level of CXCL8 in PK15 cells was detected using qRT-PCR after incubation with LPS.

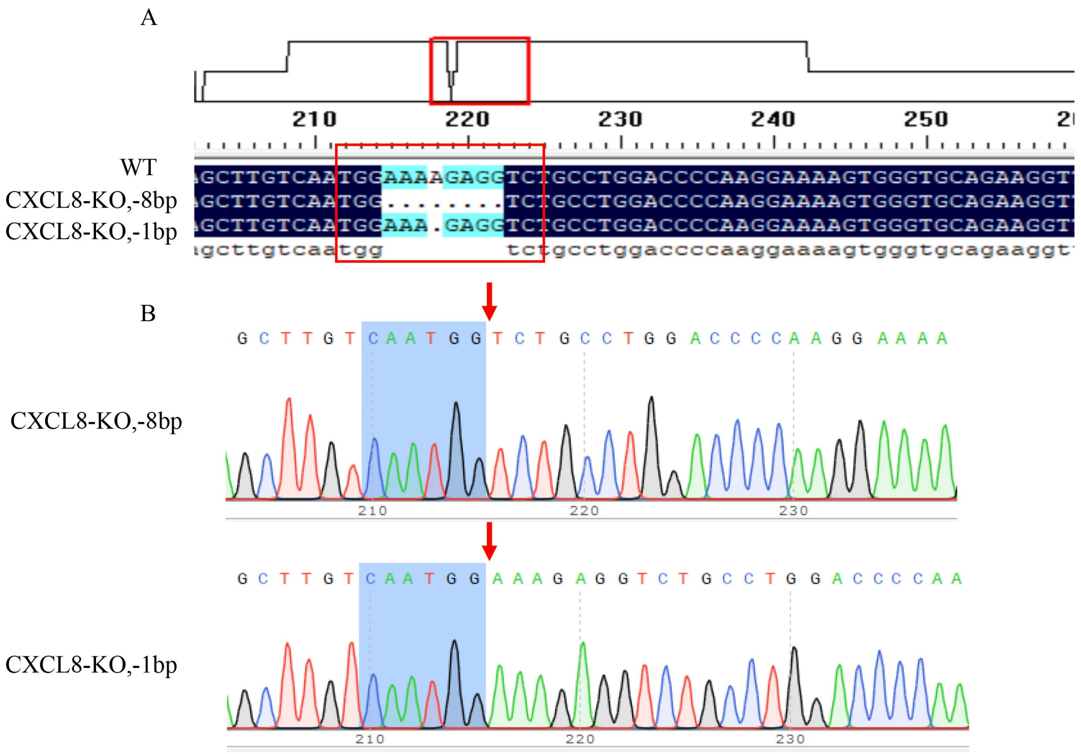

Figure S9. Identification of CXCL8-KO knockout cells

(A) Sequencing comparison of CXCL8 knockout cells and PK15 normal cells. (B) Sequencing results of CXCL8-KO knockout cells delete 8bp and 1bp respectively.

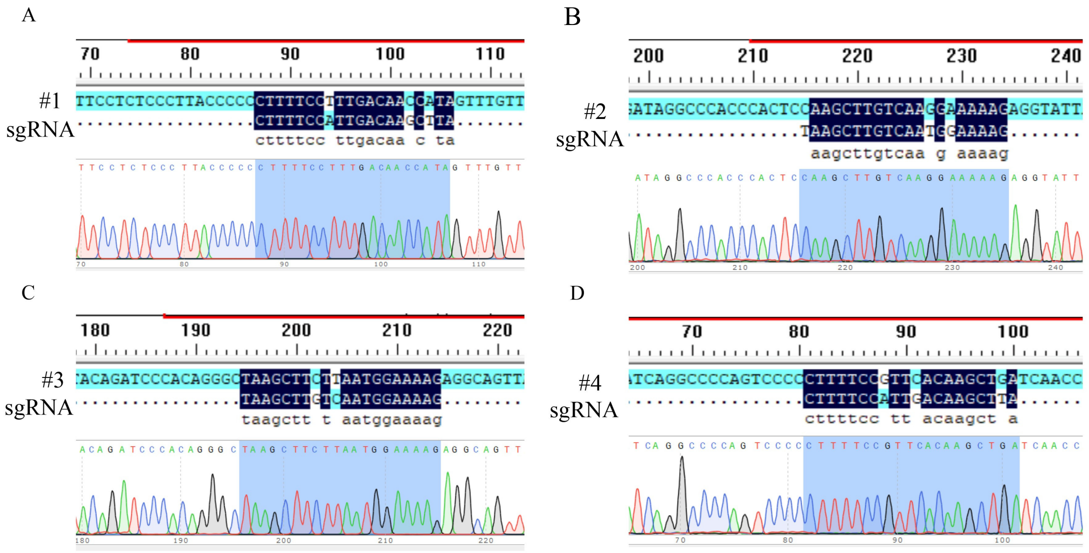

Figure S10. Identification of CXCL8-KO knockout cells Off target sites

(A) Sequencing comparison of the off target #1. (B) Sequencing comparison of the off target #2. (C) Sequencing comparison of the off target #3. (D) Sequencing comparison

of the off target #4.

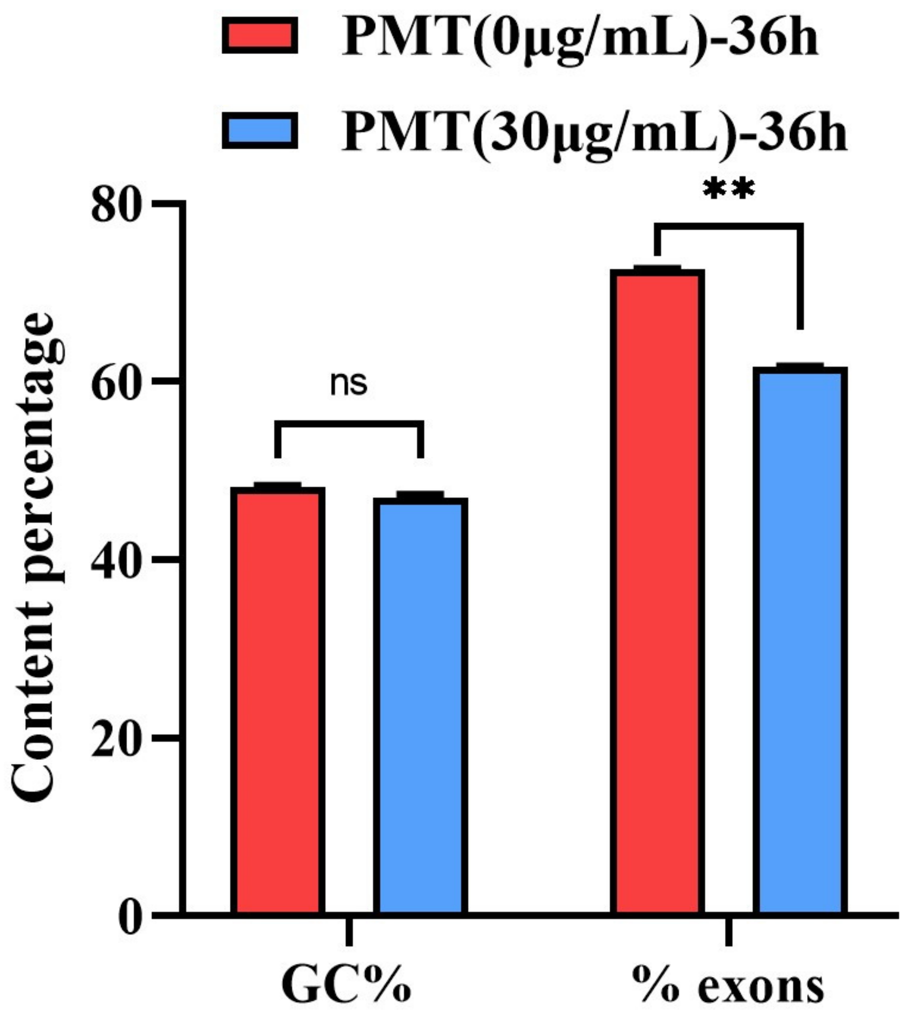

Figure S11. The percentage of GC and exons in the PK15 genome.

Table S1. Determination of endotoxin content.

Endotoxin content	OD545 nm		
1 EU/ml	0.665404	0.704213	0.708293
0.5 EU/ml	0.431858	0.450397	0.452591
0.25 EU/ml	0.326573	0.339489	0.345628
0.1 EU/ml	0.31038	0.328482	0.325156
PMT protein	0.270292333	0.270157	0.27735
negative	0.0415581	0.042467	0.041222

Table S2. Identification of Off target sites by PCR with primers.

Sequence (5′-3′)	For purpose of
F1: ATTGAGTTTAGTTCCCTGTG<br>R1: CCTGGCATTACCATGAGC	PCR for sgRNA#1
F2: TGTTGCCTTGCTTGACTA<br>R2: TCTCCTCTTGCCTGAAAT	PCR for sgRNA#2
F3: TGTTGACCCTTTCATTTCC<br>R3: GAGCATCCACAGGCAGAC	PCR for sgRNA#3
F4: CTCAGCAATGGAGGGTAT<br>R4: TGGGTTCAGCCTAACTAAC	PCR for sgRNA#4
